# Circulating microRNA responses to acute whole-body vibration and resistance exercise in postmenopausal women

**DOI:** 10.3389/fendo.2022.1038371

**Published:** 2022-11-11

**Authors:** Samuel R. Buchanan, Ryan M. Miller, Michelle Nguyen, Christopher D. Black, J. Mikhail Kellawan, Michael G. Bemben, Debra A. Bemben

**Affiliations:** ^1^ Department of Health and Human Performance, University of Texas Rio Grande Valley, Edinburg, TX, United States; ^2^ Department of Health and Exercise Science, University of Oklahoma, Norman, OK, United States

**Keywords:** microRNA, bone markers, aging, muscle mass, bone density

## Abstract

Evaluating alterations in circulating microRNA (c-miRNA) expression may provide deeper insight into the role of exercise in the attenuation of the negative effects of aging on musculoskeletal health. Currently, there are sparse data on c-miRNA responses to acute exercise in postmenopausal women. The purpose of this study was to characterize the effects of acute bouts of resistance exercise and whole-body vibration on expression of selected c-miRNAs in postmenopausal women aged 65-76 years (n=10). We also examined relationships between c-miRNAs and muscle strength and bone characteristics. This randomized crossover design study compared c-miRNA responses to a bout of resistance exercise (RE) (3 sets 10 reps 70% 1 repetition maximum (1RM), 5 exercises) and a bout of whole-body vibration (WBV) (5 sets 1 min bouts 20Hz 3.38mm peak to peak displacement, Vibraflex vibration platform). DXA was used to measure body composition and areal bone mineral density (aBMD) of the total body, AP lumbar spine, and dual proximal femur. pQCT was used to measure tibia bone characteristics (4%, 38%, 66% sites). Blood samples were collected before exercise (Pre), immediately-post (IP), 60 minutes post (60P), 24 hours (24H), and 48 hours (48H) after exercise to measure serum miR-21-5p, -23a-3p, -133a-3p, -148a-3p (qPCR) and TRAP5b (ELISA). There was a significant modality × time interaction for c-miR-21-5p expression (p=0.019), which decreased from 60P to 24H after WBV only. TRAP5b serum concentrations significantly increased IP then decreased below Pre at 24H for both WBV and RE (p<0.01). Absolute changes in TRAP5b were negatively correlated with c-miR-21-5p fold changes (r= -0.642 to -0.724, p<0.05) for both exercise modalities. There were significant negative correlations between baseline c-miRNAs and bone status variables (r= -0.639 to -0.877, p<0.05). Our findings suggest that whole-body vibration is a sufficient mechanical stimulus for altering c-miR-21-5p expression, whereas a high intensity resistance exercise protocol did not elicit any c-miRNA responses in postmenopausal women. Increases in the bone resorption marker, TRAP5b, were associated with greater downregulation of c-miR-21-5p expression.

## Introduction

Life expectancy worldwide has increased by five years between 2000 and 2016, the fastest increase since the 1960s. Unfortunately, our ability to live longer also has increased the incidence of noncommunicable diseases such as osteoporosis from low bone mass and sarcopenia due to low muscle mass. This increase in disease in the elderly has increased healthcare costs numbering in the billions of dollars (1). The current gold standard for diagnosis of osteoporosis is areal bone mineral density (aBMD) measured by dual energy x-ray absorptiometry (DXA). While DXA has high validity and precision, tracking of disease progression is limited due to the long periods of time between scans necessary to detect changes. To assess the relationship between osteoblast and osteoclast activity, it is possible to measure serum levels of bone turnover markers, though they are not currently accepted for use in diagnosing osteoporosis (2). Recently, blood assessments of microRNAs (miRNA, miR) have been suggested to be valuable markers of bone-related diseases, including osteoporosis (3).

MiRNAs are short (18-25nt), noncoding strands of RNA that influence genes post-transcriptionally. They are typically negative regulators of genetic expression by interfering with or destroying their associated messenger RNA (mRNA) target (4). Multiple RNAs are targetable by a single miRNA, and greater than 60% of human protein-coding genes are affected by them (5). Typically, only a few miRNAs are expressed for a given tissue (6) so the presence of c-miRNAs gives a basis for use as biomarkers for various tissue and physiological systems. There is evidence that deregulated miRNAs affect bone metabolism and have potential as biomarkers for osteoporosis, including miR-21-5p, -23a-3p, -124, -125b-3p, -133a-3p, -148a-3p (7) (**Supplementary Table S1**). MiR-21-5p promotes bone formation by inhibiting Small Mothers Against Decapentaplegic 7 (SMAD 7), a protein that inhibits osteoblast differentiation *via* the bone morphogenetic protein (BMP) and transforming growth factor-β (TGF-β) pathways. However, it also inhibits programmed cell death protein 4 (PDCD4) in osteoclasts, leading to increased osteoclast differentiation. MiR-23a-3p has a negative impact on bone by decreasing osteoblast differentiation. MiR-133a-3p promotes bone resorption by inhibiting the inhibitors of osteoclastogenesis. MiR-148a-3p has doubly negative impacts on bone status by increasing resorption through osteoclastogenesis and decreasing formation by inhibiting osteoblasts (7).

Aging is also associated with gradual loss of muscle mass from loss of muscle fibers and reduction in the cross-sectional area of remaining fibers and reductions in functional strength (8). The loss of muscle mass has implications, especially in the lower limbs, for increasing fall risk. Currently, there are multiple approaches for diagnosing sarcopenia with varying degrees of success in predicting falls (9). Diagnostic tests involve assessment of appendicular lean mass expressed relative to height squared by DXA, typically utilized in conjunction with a functional performance test like gait speed or muscle strength (10). Potential serum markers investigated for sarcopenia include inflammatory cytokines, anabolic hormones, and antioxidants, but these are not muscle-specific and may not reflect the physiology of skeletal muscle (11). MiR-21-5p, -23a-3p, -133a-3p, -148a-3p also have RNA targets in skeletal muscle (**Supplementary Table S1**). Targeting PDCD4 in fibers, miR-21-5p prevents myoblasts from undergoing apoptosis (12). MiR-23a-3p positively affects muscle by targeting V-maf musculoaponeurotic fibrosarcoma oncogene homolog B (MAFB) which reduces myostatin, a myokine that inhibits cellular growth (13). MiR-133a-3p has binding targets for serum response factor (SRF), which is responsible for muscle proliferation and differentiation (14). MiR-148a-3p positively affects muscle by inhibiting rho associated coiled-coil containing protein kinase 1 (ROCK1), a negative regulator of myoblast differentiation (15).

Though aging is associated with decreased muscle mass and strength (8), and increased bone loss (16), physical activity can attenuate or reverse these negative effects. *In vivo* animal models and cell culture studies have documented that mechanical loading increased bone formation rates (17) and suppressed osteoclast formation (18), and that WBV treatment inhibited osteoclast formation (19, 20). In middle-aged and older humans, chronic resistance exercise increases muscle strength (21) and improves aBMD or attenuates its loss (22, 23). Whole-body vibration (WBV) training also improves lower body muscle strength (24) and attenuates or reverses bone loss (25). Interventions combining resistance exercise and WBV have additive effects on muscle strength gains in postmenopausal women (26).

Future endeavors to reduce deterioration with aging will require a better understanding of human genes, their expression and regulation (27), and how expression can be favorably manipulated with interventions. From a genetic perspective, characterizing circulating miRNA (c-miRNA) responses to exercise is still in its infancy (28), with few published studies in older populations and none in postmenopausal women or with WBV. Exercise influences physiological processes within various systems and expression changes in c-miRNA may potentially reflect the adaptations occurring within target tissues, though there is currently limited evidence (29). Aging research specifically may benefit from this line of research, as serum sample collection is relatively non-invasive in a population where bone and muscle tissue collection may be difficult (29). With the ability of c-miRNA to be taken up by tissues (30), expression in serum may also be indicative of a functional role in adaptations to exercise. However, the paucity and inconsistent results in recent literature characterizing c-miRNA and exercise responses make their use as exercise biomarkers limited at present (31). More exercise studies, conducted in a variety of populations using various modalities, are needed to fully characterize c-miRNA expression responses to exercise before they can be reliably used as biomarkers (31). Therefore, the primary purposes of this study were to: (1) characterize the effects of acute bouts of resistance exercise (RE) and whole-body vibration (WBV) on expression of selected c-miRNAs in postmenopausal women aged 65-76 years; and (2) determine whether exercise responses of c-miRNA and the bone resorption marker, tartrate-resistant acid phosphatase 5b (TRAP5b) are correlated. Based on their biological effects on bone and muscle tissue, it was hypothesized that c-miRNA (c-miR-21-5p, -23a-3p, -133a-3p, -148a-3p) expression would increase immediately post exercise for both protocols as a result of plasma volume shifts, followed by downregulation at 24 hours and 48 hours post exercise. TRAP5b absolute changes would be positively correlated with c-miR-133a-3p and -148a-3p fold changes and negatively correlated with c-miR-21-5p and -23a-3p fold changes.

## Methods

### Participants

Fourteen participants were initially enrolled in the study; 4 participants were excluded prior to testing due to voluntary termination (n=2), injury outside of the study (n=1), and an inability to give blood draws (n=1). In total, 10 participants, 65-76 years of age were used in the final analysis. Inclusion criteria were: women 60–85 years of age; independent living; greater than 5 years postmenopausal. Exclusion Criteria were: current smokers; individuals with metabolic disease (e.g., diabetes), cancer, or uncontrolled hypertension; individuals taking medications, other than bisphosphonates, known to affect bone metabolism, such as hormone replacement therapy, antidepressants, or glucocorticoids; individuals who had sustained a fracture within the previous 12 months; and individuals with metal implants or joint replacement at the hip or spine. Participants provided written informed consent and this study was approved by the University of Oklahoma Health Sciences Center (OUHSC) Institutional Review Board (IRB#9569)

### Research design

This within-subjects randomized crossover study design for RE and WBV conditions required 9 visits: consenting, blood pressure, and questionnaires (visit 1); bone scans, familiarization of resistance exercise equipment and whole body vibration, and functional performance measures of handgrip strength and jump power (visit 2); 1 repetition maximum (1 RM) testing of leg press, shoulder press, latissimus (lat) pulldown, leg extension, and hip adduction (visit 3); resistance exercise testing (visit 4); whole body vibration testing (visit 7); Visits 4 and 7 were randomized with a minimum of a 10-day washout period between exercise visits. Visits 5, 6, 8, and 9 consisted of blood draws that occurred between 8:00-9:00 a.m. 24 and 48 hours after each exercise visit. Medical clearance was obtained before Visit 2. Study visits are illustrated in **Supplementary Figure S1**.

An *a priori* power analysis was performed with G*Power 3.1.9.2 software based on the study by Daniels et al. (32) with power set at 0.8, alpha at 0.05 for detecting changes in blood miRNA expression over 5 timepoints. Calculated effect sizes were 0.34 and 0.41, requiring sample sizes of 10 to 12 for 80% power.

### Questionnaires

Questionnaires were completed by participants to determine inclusion/exclusion criteria and collect information to reduce the potential confounding influence of physical activity, diet, and menstrual history. An in-house health status questionnaire was used to determine if participants meet study criteria and if they had any preexisting conditions that warranted exclusion and used to record medications taken by the participants. An in-house menstrual history questionnaire provided information about menstrual history and any hormone replacement therapy. Physical activity status was determined by the International Physical Activity Questionnaire (IPAQ) designed to designate low (<600 MET min/week), moderate (≥600 to<3,000 MET min/week), or high (≥3,000 MET min/week) physical activity levels per week (33), and the Bone-Specific Physical Activity Questionnaire (BPAQ) that quantified exposure to bone loading physical activity throughout the lifespan (34). Daily calcium Intake was estimated from a food frequency questionnaire that included supplements (35).

### Anthropometric measurements and blood pressure

Height was measured to the nearest 0.5 cm using a wall stadiometer (PAT #290237, Novel Products, Rockton, IL). Weight was measured to the nearest 0.1 kg with a digital electronic scale (BWB-800, Tanita Corporation of America, Inc., Arlington Heights, IL). Participants’ resting blood pressure was measured with an automatic blood pressure monitor (Omron, Japan) on the left arm. Participants with values above 140 mmHg systolic or 90 mmHg diastolic pressure were excluded from further study participation.

### Dual energy X-ray absorptiometry

The Lunar Prodigy DXA (GE Healthcare, enCORE software, version 16, Madison, MI) was used to measure areal bone mineral density (aBMD) and body composition. The four scan sites were the total body, AP lumbar spine (L1-L4), and dual proximal femur (femoral neck, trochanter, and total hip). Total body scans provided body composition of the whole body and appendicular skeletal muscle mass (ASMM) for potential sarcopenia classification. Participants were instructed to remove jewelry, shoes, and lie supine and centered on the scanning table with the top of their head approximately 2-3 cm below the horizontal white line for the total body scan. Hips and shoulders were adjusted, as necessary, to position the participant evenly in the middle of the scanning field. Straps were used below the knee and at the ankles to maintain leg positioning. For the spine scan, the legs were raised, and a foam block placed underneath so the knees were bent at a 45-to-60-degree angle. The dual femur scan required the foam block and straps to be removed. A brace was placed between the ankles and strapped in place. The left femur was positioned directly parallel with the table, then the right femur. Scans were analyzed with encore software, v16 (GE Healthcare, Madison, WI). Quality Assurance (QA) tests were performed and documented before each scanning day for calibration of the device. *In vivo* precision (RMS CV%) for aBMD for our lab are 1.27% for total body, 1.8% for AP spine, and 1.0 – 1.79% for hip variables. For soft tissue variables, RMS CV% are 1.21% for bone-free lean body mass, 1.74% fat mass, and 2.08% for appendicular skeletal muscle mass. Hydration status was determined with a urine refractometer (VEE GEE CLX-1, Rose Scientific Ltd., Alberta, Canada). Acceptable hydration for body composition determination is a urine specific gravity between 1.004-1.029 (36).

### Peripheral quantitative computed tomography

An XCT-3000 bone scanner (Stratec Medizintechnik GmbH, Pforzheim, Germany) was used for the epiphyseal and diaphyseal bone measurements of the non-dominant tibia at the 4%, 38%, and 66% sites. Integrated software v6.00 (Stratec Medizintechnik GmbH, Pforzheim, Germany) was used for analysis. Prior to scanning, non-dominant tibia length (mm) was assessed using a tape measure. Participants were instructed to sit in a chair and cross their non-dominant leg over their dominant knee. Scans were obtained with a 0.4 mm voxel size, 2.2 mm slice, and a 20 mm/s scan speed. At the distal tibia (4%), cont mode 3 at 169 mg/cm3 and peel mode 4 at 650 mg/cm3 with a 10% peel were used to determine total vBMD (mg/cm3), total bone area (mm2), trabecular vBMD (mg/cm3), trabecular area (mm2), and bone strength index (BSI) (mg/mm4). For 38% and 66% tibia, cont mode 2 at 710 mg/cm3 was used to define total vBMD (mg/cm3), total bone area (mm2), cortical vBMD (mg/cm3), cortical area (mm2), cortical thickness (mm), while cont mode 2 at 480 mg/cm3 was used to obtain resistance to torsional deformation polar moment of inertia (Ipolar) (mm4) and torsional polar strength for strength-strain index (pSSI) (mm3). Muscle cross-sectional area (MCSA) (mm2) was also calculated for the 66% tibia site. RMS CV% for all pQCT variables for our lab range from 0.68 – 3.07% for the 4% site, 0.29 – 0.61% for the 38% site, and 0.49 – 1.85% for the 66% site.

### Strength testing

Leg press, shoulder press, lat pulldown, leg extension, and hip adduction isotonic machines (Cybex, Medway, MA) were used for this study. Trained personnel were present to instruct participants on appropriate lifting technique. Participants warmed up for 5 min at a self-selected comfortable pace and resistance on a stationary bicycle (828E, Monark, Vansbro, Sweden). The 1RM protocol for each piece of equipment was: (1) proper positioning based on manufacturer recommendations; (2) complete a warmup set of 5-10 repetitions at ~50% of estimated maximal strength; (3) after 1 min rest, another set of 3-5 repetitions at ~75% of estimated maximal strength; (4) After 2 min rest, the load was increased for 1 repetition, with this step repeating, until a maximum was achieved. Maximum strength was achieved within 5 attempts.

### Acute exercise protocols

Participants performed 2 upper body and 3 lower body resistance exercises in the following order: leg press, shoulder press, lat pulldown, leg extension, and hip adduction. There were 3 sets of 10 repetitions per exercise at 70-75% of 1RM with 2-3 min of rest between sets and exercises. Higher intensities were chosen based on the safety considerations and effectiveness of improving muscle and bone strength in an elderly population (37). Each repetition consisted of ~1 second each during the eccentric and concentric phases, with minimal time spent isometrically. One participant failed at 8/10 repetitions on the third set of shoulder press and the weight was reduced by 2.5kg to complete the last two repetitions. All other participants completed every repetition for all sets and exercises at the prescribed load.

The WBV protocol required participants to stand barefoot with knees bent at 30° and their second toe in line with the dot located between positions 1 and 2 on the Vibraflex Vibration Platform (Orthometrix, Inc., Naples, FL). Each of the 5 bouts were performed for one min at a 20 Hz frequency with a 3.38 mm peak-to-peak displacement and a peak acceleration for each vibration bout of approximately 2.7g and 1 min of rest between bouts to restore mechanosensitivity to bone cells (38). Peak acceleration was calculated from the following formula for side-alternating vibration platforms: G-Force= (A(2πf)2)/9.81 (39). The knee-bent positioning with high amplitude and low frequency on an oscillating platform has been shown through meta-analysis to be the most effective protocol for stimulating bone formation (25). Four participants reported minor side effects from the WBV exposures, including itchiness (n=2), dizziness (n=1), and low back tightness (n=1).

### Blood sampling

Blood samples (8.5 ml) were collected *via* venipuncture by a registered phlebotomist. Sampling times are illustrated in **Supplementary Figure S2**. Baseline samples (Pre) on exercise days were collected at 8:00 am after an 8h overnight fast to measure c-miRNA and TRAP5b, with further sampling immediately (IP), 60 minutes (60P), 24 hours (24H), and 48 hours (48H) post-exercise. Blood draws at 24H and 48H occurred between 8-9:00 am. One participant did not go to the clinic for her 24-hour blood draw, thus n=9 for that timepoint. For the exercise day blood draws, two hematocrit tubes were filled from the serum separator tubes for measurement of hematocrit (HCT) and plasma volume shifts. Percent change in plasma volume (%ΔPV) was determined with the following equation:


%ΔPV = 100/(100 – HCT Pre) * 100((HCT Pre – HCT Post)/HCT Post)

(40) and applied to TRAP5b concentrations with the following equation:


Corrected Concentration = Uncorrected value * ((100 + %ΔPV)/100

Blood lactate was analyzed with the Lactate Plus lactate analyzer (Sport Resource Group Inc., Minneapolis, MN) pre and immediately post-exercise. Blood samples then were allowed to clot for 30 min and centrifuged at 2,000 g for 15 min to obtain serum, which was were equally aliquoted into 8 microtubes and frozen at -84°C until analysis.

### TRAP5b assays

MicroVue™ Commercial EIA kits were used to measure TRAP5b (Quidel, Athens, OH) in duplicate. All assays were performed following step by step instructions included with each kit. Intra-assay CV% ranged from 1.3-7.9% and the inter-assay CV% was 7.2%.

### MicroRNA quantification

Target miRNAs (miR-21-5p, -23a-3p, -133a-3p, -148a-3p) were selected based on their regulatory functions on bone metabolism and muscle tissue (7, 41) (**Supplementary Table S1**). MiRNA analyses were performed by TAmiRNA GmbH miRNA quantification service (Vienna, Austria). Total RNA was extracted using the miRNeasy Mini Kit (Qiagen, Hilden, Germany) following procedures included in the kit. Standardized methods and quality controls were performed according to the Minimum Information for Publication of Quantitative Real-Time PCR Experiment Guidelines (MIQE) (42) to ensure quality assurance for the miRNA workflow. The reactions were run in duplicate. Serum samples were thawed on ice and centrifuged at 12,000g for 5 min for cellular debris removal. 200µl of serum were used for sample lysis by mixing with 1,000µl QIAzol Lysis Reagent and 1µl of synthetic spike-in, Uni-Spike-In 4 (UniSp4) (Exiqon, Vedbaek, Denmark), to control for variance of cDNA synthesis and qPCR. After incubation at room temperature for 10 min, RNA extraction was performed using 200µl chloroform and phase separation achieved by centrifugation at 12,000g for 15 min at 4°C. 650µl of the upper aqueous phase was transferred to a new collection tube and mixed with 7µl glycogen. Samples were transferred to an miRNeasy mini column, and RNA was precipitated with 750µL of ethanol followed by washing with RPE and RWT buffer. RNA was eluted in 30µL of nuclease-free water and stored at −80°C until further analysis. Detection of hemolysis was performed using the Nanodrop OD414 measurement. Spike-in controls were used for quality control: 1. Uni-Spike-In 4 (UniSp4) to monitor RNA extraction efficiency and assess the overall variability of the assay; and 2. Cel-miR-39-3p was added after RNA isolation to monitor the presence of inhibitors and variability during cDNA synthesis and qPCR. The CV% were 1.62% for the UniSp4 RNA spike-in and 1.32% for the cel-miR-39-3p cDNA spike-in.

### cDNA synthesis

The extracted RNA was transcribed to cDNA using the Universal cDNA Synthesis Kit II (Exiqon). The protocol was modified in that 2µL of total RNA was used per 10 µL reverse transcription (RT) reaction. Polymerase chain reaction amplification was performed in a 96-well plate format using custom Pick-&-Mix plates (Exiqon) in a Roche LC480 II instrument (Roche, Mannheim, Germany) and EXiLENT SYBR Green Master Mix (Exiqon) with the following settings: 95°C for 10 min, 45 cycles of 95°C for 10 seconds and 60°C for 60 seconds, followed by melting curve analysis.

### Quantification of miRNA expression

Quantification of expression was determined by assessing the quantification cycle (Cq) of selected c-miRNA utilizing the 2nd derivative method (43). Quality control data for RNA spike-ins are provided in **Supplementary Figure S3**. Data was normalized to the RNA spike-in control (UniSP4) that was added in before RNA extraction with the equation Cq = Cq(UniSP4) – Cq(miRNA). The 2-ΔΔCt was used to calculated fold changes from pre values (44 **).**

The following formula was used to adjust miRNA expression responses for plasma volume changes with an alteration to the formula to account for exponential expression changes in quantification cycle (Cq) values for miRNA expression with the formula %ΔPV = (log(100)/log(100) – log(HCT Pre)) * log(100)* ((log(HCT Pre) – log(HCT Post))/log(HCT Post)). The correction factor was subtracted from the Cq values for each miRNA from each exercise sample.

### Data analyses

Statistical analyses were performed using IBM SPSS Statistics (SPSS Inc., Chicago, IL), version 24. Relative expressions of c-miRNA are reported as mean ± standard error (SE), with other descriptive data reported as mean ± standard deviation (SD). Normality of dependent variables was assessed *via* Shapiro-Wilk tests. For participant characteristics, bone and muscle variables, normality was only violated for current BPAQ scores, IPAQ, and 66% total volumetric bone mineral density. For miRNAs, all pre-exercise log2-tranformed values were normally distributed. The 24H timepoint on the RE day for miR-21-5p was non-normally distributed, as well as WBV miR-133a-3p 48H and RE miR-133a-3p IP; however, the data for each timepoint followed normal straight-line Q-Q plots. Separate Kruskal-Wallis tests were run for separate modalities to see differences between timepoints and Mann-Whitney U tests to compare timepoints to pre-exercise expression. Paired t-tests were performed between pre-exercise WBV and RE for c-miRNAs.

Two-way mixed-model repeated measures ANOVA [modality × time] were used to assess changes across time for c-miRNAs, lactate, and TRAP5b between the two exercise modalities. ANOVAs included modality × time for all five timepoints (Pre, IP, 60P, 24H, 48H), and separate ANOVAs for the 3-exercise day timepoints (pre, IP, 60P) and recovery days (Pre, 24H, 48H) for the c-miRNA. For significant modality × time interactions, one-way ANOVAs across time within each modality with Bonferroni corrections and paired t-tests between modalities at each timepoint were used for *post-hoc* pairwise comparisons. Pre-exercise data was set as the control to calculate fold change of c-miRNA expression for subsequent timepoints (IP, 60P, 24H, 48H). Pearson’s correlation coefficients were utilized for normally distributed variables and Spearman’s rank rho for the four non-normally distributed variables to determine associations between microRNAs and muscle strength, bone variables, and TRAP5b. MiRNA fold change (FC) effect sizes for differential expression were ≥ 2 for upregulation and ≤ 0.5 for down regulation. Effect sizes for the two-way ANOVA results are reported as partial eta squared (ηp2) with small, medium, and large effect sizes indicated by ηp2 values of 0.0099, 0.0588, and 0.1379, respectively (45). The level of significance was set at p ≤ 0.05.

## Results

### Participant characteristics

Participant characteristics and physical performance measures are found in **Table 1** and **Supplementary Table S2**. Calcium intake ranged from 270-1,690 mg/day, with the mean above the recommended 1,000mg/day (46). Additionally, 8 participants were considered highly active according to the IPAQ questionnaire with 2 classified as moderately active (33). According to the European Working Group on Sarcopenia in Older People (EWGSOP) 2018 criteria (ASMM)< 15kg or ASMM/height^2^< 5.5kg/m2) (47), 2 participants were sarcopenic based on ASMM alone, but none had ASMM/height^2^ values below 5.5kg/m2. **Supplementary Table S3** shows the DXA variables for the total body, spine, and dual femur scans. Assessment of bone status by T-scores (48), determined 1 participant was osteoporotic, 7 were osteopenic, and 2 had normal bone status. **Supplementary Table S4** depicts pQCT variables for the 4%, 38%, and 66% non-dominant tibia sites, respectively.

**Table 1:** Participant characteristics (n=10) (means ± SD).

Age (yrs)	70.6	±	4.27
Height (cm)	159.6	±	6.19
Body Mass (kg)	64.51	±	12.47
Total Body Fat %	36.93	±	7.44
Total Body Fat Mass (kg)	24.48	±	8.54
Total Body BFLBM (kg)	37.89	±	4.75
Total Body BMC (g)	2068.0	±	187.1
ASMM (kg)	16.23	±	2.66
ASMM/height^2^ (kg/m^2^)	6.33	±	0.69
Osteoporosis/Osteopenia (%)	10%	/	70%
Calcium Intake (mg/day)	1174	±	446
BPAQ Past	58.3	±	28.1
BPAQ Current	4.2	±	10.3
BPAQ Total	31.3	±	15.1
IPAQ MET-min/week	6296	±	5670

BFLBM, Bone Free Lean Body Mass; BMC, Bone Mineral Content; ASMM, Appendicular Skeletal Muscle Mass; BPAQ, Bone-Specific Physical Activity Questionnaire; IPAQ, International Physical Activity Questionnaire; MET, Metabolic Equivalent.

### Lactate and plasma volume

**Table 2** displays exercise responses for plasma volume changes and lactate concentrations for the pre, immediate post (IP), and 60 min post-exercise (60P) timepoints. For lactate there was a significant modality × time interaction (p<0.001) and significant main effects for modality (p=0.01) and time (p<0.001). *Post hoc* comparisons showed an increase in lactate pre to IP for WBV (p=0.027) and RE (p=0.001) but only RE had higher lactate concentrations from IP to 60P (p=0.002). For plasma volume change, there was no significant interaction or modality difference, but there was a significant effect for time with the plasma volume decrease being greater for IP than 60P (p=0.041).

**Table 2:** Lactate and plasma volume changes (means ± SD).

	Time	WBV (n = 10)			RE (n = 10)		
Lactate (mmol/L) ^§†^	Pre		0.72 ± 0.23*			0.76 ± 0.32*
	IP		1.11 ± 0.47			2.73 ± 1.47	
	60P		0.80 ± 0.42			0.84 ± 0.37*	
PVΔ (%)	IP		-3.48 ± 4.6			-8.55 ± 3.89	
	60P		-3.57 ± 6.69*			0.19 ± 7.78*	

PVΔ, Plasma Volume Change; WBV, whole-body vibration; RE, resistance exercise.

§p ≤ 0.001 significant modality × interaction.

†p ≤ 0.05 modality difference.

*p ≤ 0.05 time difference from IP.

### Bone resorption marker

There were no significant modality or modality × time interaction effects, but the time main effect was significant (p<0.001) for serum TRAP5b concentrations (**Table 3**). Effect sizes were medium for modality (ηp2 0.064) and the modality × time interaction (ηp2 0.117) and large for time (ηp2 0.736). TRAP5b significantly increased from Pre to IP (p=0.048) and decreased Pre to 24H post (p=0.007) for both modalities. Also, 24H post was significantly lower than both IP (p=0.001) and 60P (p=0.003). After correcting for plasma volume shifts, there was still a significant main effect for time (p=0.008) with TRAP5b concentrations decreasing from Pre to 24H post (p=0.007).

**Table 3:** Serum TRAP5b responses (means ± SD).

	Time	WBV (n = 9)			RE (n = 9)		
TRAP5b (µ/L)	Pre		3.68 ± 1.04			3.74 ± 1.02	
	IP^*†^		3.80 ± 0.99			3.98 ± 1.10	
	60P^†^		3.72 ± 1.11			3.86 ± 1.04	
	24H^*^		3.40 ± 0.97			3.60 ± 1.01	
Corr TRAP5b (µ/L)	IP		3.73 ± 1.09			3.77 ± 1.01	
	60P		3.69 ± 1.16			3.56 ± 1.77	
	24H		3.40 ± 0.97			3.60 ± 1.01	
% Change vs. Pre	IP^†^		4.00 ± 9.92			6.70 ± 6.85	
	60P^†^		1.30 ± 5.81			3.56 ± 6.50	
	24H		-7.56 ± 5.27			-3.44 ± 4.33	
Corr % Change	IP^†^		0.89 ± 11.22			-0.67 ± 10.19	
	60P		-1.67 ± 9.94			-6.70 ± 33.72	
	24H		-7.56 ± 5.27			-3.44 ± 4.33	

TRAP5, Tartrate-resistant acid phosphatase 5b; WBV, whole-body vibration; RE, resistance exercise; Corr, Corrected for plasma volume change; Pre, pre-exercise; IP, immediately post-exercise; 60P, 60 minutes post-exercise; 24H, 24 hours post-exercise.

*p ≤ 0.05 significant vs. Pre.

†p ≤ 0.05 significant vs. 24H.

There were no significant modality or modality × time interaction effects, but there was a significant time effect (p<0.001) for TRAP5b percent changes from Pre. The effect sizes for modality (ηp2 0.086) and time (ηp2 0.773) were similar to the raw concentration analysis, however, the modality × time interaction effect size was small (ηp2 0.022). TRAP5b percent change was significantly different at 24H compared to IP (p=0.001) and 60P (p=0.001) and after correcting for plasma volume shifts, there was still a significant difference at 24H post compared to IP (p=0.035).

### MiRNA responses

In total, 99 samples from the ten participants were analyzed for four miRNAs. MiR-21-5p was expressed in 99/99 samples (mean Cq=25.0). MiR-23a-3p was expressed in 99/99 samples (mean Cq=26.28). MiR-133a-3p was expressed in 98/99 samples (mean Cq=33.77). MiR-148a-3p was expressed in 99/99 samples (mean Cq=29.18).

**Figure 1:**
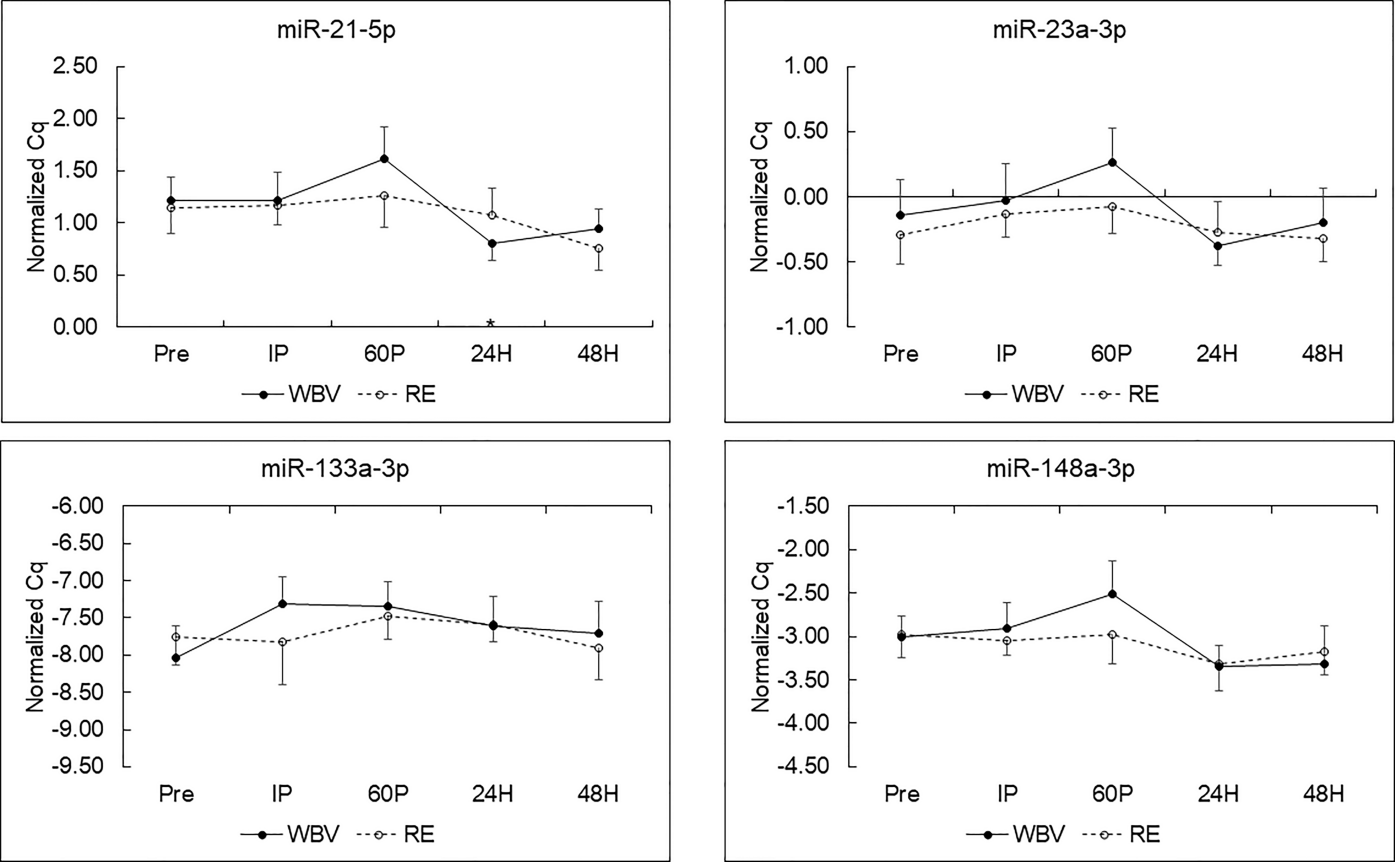
C-miRNA responses to resistance exercise (RE) and whole-body vibration (WBV) protocols (means ± SE) in postmenopausal women. *p < 0.05 vs. 60P for WBV; n=9 for miR-21-5p, -23a-p and -148a-3p; n=8 for miR-133a-3p.

MiRNA expression and effect sizes for the WBV and RE conditions are shown in **Figure 1** and **Table 4**, respectively. There were no significant differences between WBV and RE for pre-exercise c-miR-21-5p, -23a-3p, -133a-3p, or -148a-3p (all p≥0.599). Based on the 5 time point repeated measures ANOVA, c-miR-21-5p was the only miRNA to show a significant response to the exercise protocols, although the majority of the effect sizes were medium to large for all c-miRNAs. There was a significant modality × time interaction (p=0.019) for c-miR-21-5p, which decreased 60P to 24H (p=0.036) for WBV but not for RE. There were no main effects or interaction effects for any of the miRNAs when analyzed for the 3 exercise time points or comparing the resting miRNA expressions over the 3 days (pre, 24H, 48H). None of the mean fold changes in miRNAs met the criteria for differential expression (≥ 2 for upregulation and ≤ 0.5 for down regulation) (**Table 5**). Adjusting miRNA expression and fold changes for plasma volume shifts had little effect on the results (**Supplementary Tables S6**, **S7**).

**Table 4:** Circulating miRNA relative expression two-way repeated measures ANOVA effect sizes.

Effect Sizes (η_p_^2^)						
miRNA	Modality	p	Time	p	Modality × Time	p
miR-21-5p	0.047	0.546	0.197	0.125	0.300	0.019
miR-23a-3p	0.090	0.399	0.132	0.325	0.156	0.233
miR-133a-3p	0.174	0.231	0.101	0.379	0.084	0.427
miR-148a-3p	0.043	0.563	0.107	0.397	0.152	0.244

η_p_^2^ , partial eta squared.

**Table 5:** Circulating miRNA fold changes (vs. Pre).

Fold Change Means (minimum - maximum)				
miRNA	IP	60P	24H^a^	48H
miR-21-5p				
WBV	0.94 (0.59-1.51)	1.31 (0.68-2.53)	0.75 (0.44-1.27)	0.82 (0.45-1.50)
RE	1.03 (0.66-1.60)	1.04 (0.71-1.52)	1.04 (0.60-1.81)	0.70 (0.36-1.37)
miR-23a-3p				
WBV	1.07 (0.65-1.79)	1.32 (0.73-2.36)	0.85 (0.42-1.73)	0.96 (0.43-2.18)
RE	1.12 (0.77-1.61)	1.12 (0.77-1.61)	1.04 (0.70-1.54)	0.90 (0.53-1.52)
miR-133a-3p				
WBV	1.65 (0.80-3.42)	1.60 (0.72-3.53)	1.33 (0.55-3.23)	1.10 (0.67-1.82) ^b^
RE	0.95 (0.32-2.81)	1.54 (0.65-3.64)	1.33 (0.52-3.39)	0.79 (0.25-2.47)
miR-148a-3p				
WBV	1.08 (0.64-1.79)	1.42 (0.67-2.98)	0.79 (0.36-1.72)	0.81 (0.31-2.11)
RE	0.95 (0.57-1.61)	1.00 (0.61-1.65)	0.93 (0.46-1.89)	0.87 (0.69-1.96)

^a^n,9 for this time point; ^b^n,8 for this time point; WBV , whole-body vibration; RE , resistance exercise; IP , immediately post-exercise; 60P , 60 minutes post-exercise; 24H , 24 hours post-exercise; 48H, 48 hours post-exercise.

**Figure 2:**
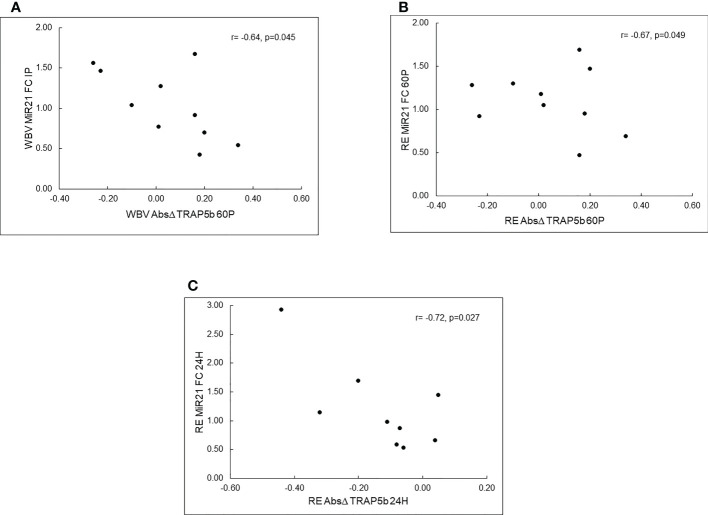
Pearson correlations between c-miRNA fold changes (FC) and TRAP5b absolute changes (Δ) for resistance exercise (RE) and whole-body vibration (WBV). Panel **(A)**–WBV c-miR-21-5p FC at IP vs. WBV TRAP5b Absolute Δ at 60P, Panel **(B)** – RE c-miR-21-5P FC at 60P vs. RE TRAP5b Absolute Δ at 60P, Panel **(C)**-RE c-miR-21-5p FC at 24H vs. RE TRAP5b Absolute Δ at 24H; IP - immediately post-exercise; 60P-60 minutes post-exercise; 24 hours post-exercise.

MiR-21-5p fold changes were negatively associated with absolute changes in TRAP5b for both exercise protocols (**Figures 2A-C**). Specifically, miR-21-5p FC IP was negatively correlated with absolute change in TRAP5b 60P (r=-0.642, p=0.045) for WBV, miR-21-5p FC 60P was negatively correlated with absolute change in TRAP5b 60P (r=-0.668, p=0.049) for RE, and miR-21-5p FC at 24H was negatively correlated with absolute change in TRAP5b at 24H (r=-0.724, p=0.027) for RE.

As shown in **Supplementary Table S5**, there were many significant correlations between baseline miRNA expression values (averaged over both WBV and RE test days) and bone characteristics. TRAP5b was significantly positively associated with miR-133a (r=0.758, p=0.01). Of note, miR-21-5p was significantly negatively correlated with tibia variables (r=-0.658 to -0.877, all p<0.05), miR-23a-3p was significantly correlated with left total hip BMD (r= -0.642, p=0.046) and 38% cortical area (r=-0.639, p=0.047), miR-133a-3p was positively correlated L1-L4 aBMD (r=0.666, p=0.036), and TRAP5b pre (r=0.758, p=0.011), and miR-148a-3p was significantly negatively correlated tibia variables (r=-0.635 to -0.821, all p<0.05).

## Discussion

To our knowledge, this is the first study to investigate c-miRNA responses to acute bouts of resistance exercise and whole-body vibration in postmenopausal women. Unique findings were that c-miR-21-5p expression decreased 24 hours after WBV and miR-21-5p fold changes were negatively correlated with post-exercise absolute changes in TRAP5b for both exercise modalities. This is also the first known study to attempt to correct c-miRNA expression changes that may be due to plasma volume shifts. Serum TRAP5b concentrations increased immediately post-exercise then decreased below baseline by 24 hours for both WBV and RE, although the increase in TRAP5b immediately post-exercise was accounted for by plasma volume shifts. Generally, baseline c-miRNA expression was negatively associated with measures of bone metabolism but not with muscle variables.

Our findings support that miR-21-5p expression is responsive to mechanical stress imposed on the musculoskeletal system by WBV. Mechanical loading has been shown to change miRNA expression, which in turn, may be an important regulatory mechanism for the exercise-induced adaptations in bone metabolism (49). The lack of miRNA response to RE was unexpected and the underlying reasons for the different responses to WBV and RE are not clear. One possible explanation is that WBV poses a novel stimulus to bone in these physically active women. Also, the type of stresses imposed on the bone (e.g., shear stress, compression, torsional) may differ between WBV and RE. Cell line studies provide evidence that miR-21-5p is sensitive to mechanical loading as it was up-regulated in human periodontal stem cells by a stretch load (50) but down-regulated in MC3T3-E1 cells by fluid shear stress promoting osteogenic differentiation (51). In mice, miR-21-5p deficiency inhibited osteoclast function and bone resorption leading to increased trabecular bone accrual (52). Several clinical studies (53, 54) found that both serum and bone tissue miR-21-5p expression was higher in patients with osteoporotic fractures compared to normal controls. Taken together, these results suggest the c-miR-21-5p decrease at 24 hours post-WBV we observed may be a favourable response for enhancing bone mass over time in postmenopausal women.

In addition to effects on bone, the miRNAs we selected have been shown to regulate skeletal muscle development (40, 55). MiR-21-5p, when overexpressed in denervated muscles, leads to enhanced muscle atrophy through targeting of YY1, eIF4E3, and PDCD10 (56). In mice, miR-23a-3p was shown to attenuate skeletal muscle atrophy through targeting of MAFbx/atrogin-1 (57). MiR-133a-3p targets SRF in muscle, leading to increased myocyte proliferation and miR-148a-3p promotes myogenic differentiation (40). Although we detected significant mean changes only for c-miR-21-5p, individual participants had biologically significant differential expression, such as upregulation (≥ 2 FC), while others showed downregulation (≤ 0.5 FC) for a given miRNA. Presently, the mediating factors that would explain these disparate responses are not clear.

Contrary to our hypothesis, the acute bout of resistance exercise did not elicit any changes in c-miRNA expression in our cohort of postmenopausal women. This lack of response contradicts previous reports of altered c-miRNA expression after acute bouts of RE in young men (58, 59). The type of resistance exercise protocol may modulate the c-miRNA responses. Cui et al. (58) compared c-miRNA expression to single bouts of maximal strength (4 sets, 6 reps, 90%1RM), muscular hypertrophy (3 sets, 12 reps, 70% 1RM), and strength-endurance protocols (3 sets, 15-20 reps, 40% 1RM) and found that c-miR-21-5p expression decreased immediately post exercise then returned to baseline levels by 60 minutes post exercise only for the muscular hypertrophy protocol. C-miR-133a-3p expression decreased immediately post both the muscular hypertrophy and maximal strength protocols. Although our resistance exercise protocol was similar to the muscular hypertrophy protocol used by Cui et al. (58), it did not stimulate c-miRNA responses in our postmenopausal women. Age and training status also affect miRNA profiles and responses to exercise. Age-associated declines in miRNA responses to an exercise stimulus may be partially attributed to dysregulation of miRNA expression. Margolis et al. (60) reported different patterns of c-miRNA expression in response to acute high intensity resistance exercise with aging as c-miRNA profiles were downregulated in older men but upregulated in younger men. Regarding training status, skeletal muscle miRNAs have been shown to be differentially expressed in power lifters compared to healthy controls as miR-133a-3p was lower and miR-23a-3p was elevated in powerlifters (61). C-miRNA resting profiles also were differentially expressed based on training status in young men (62) and women (63). The majority of our participants self-reported as being highly active, with 1 reporting exercising with light weights; however, they were not resistance trained nor had any experience with WBV. Given their older age and physical activity status, the c-miRNA responses of our participants to resistance exercise may have been blunted.

TRAP5b, a marker of osteoclast cell number and bone resorption (64), significantly increased post-exercise then decreased below pre-exercise levels 24 hours later for both exercise modalities. Previously, we reported similar patterns of TRAP5b responses to single bouts of resistance exercise alone or combined with WBV exposures in young adults (65, 66). The TRAP5b decrease 24 hours post exercise suggests that bone resorption was decreased, which is supported by cell culture studies showing that osteoclast formation is inhibited by mechanical loading (18), and WBV treatment (19, 20). We hypothesized that TRAP5b would be positively associated with c-miR-133a-3p and -148a-3p since these miRNAs promote osteoclast differentiation (7). While baseline TRAP5b and c-miR-133a-3p were strongly positively correlated, absolute changes in TRAP5b were negatively correlated with c-miR-21-5p fold changes for both exercise modalities, thus increases in TRAP5b were associated with downregulation of c-miR-21-5p. The potential biological meaning of these relationships is not clear since miR-21-5p promotes both osteoblast and osteoclast differentiation through different target genes and signalling pathways. However, in a recent 30-day bedrest study, we documented that c-miR21-5p was upregulated, and c-miR-21-5p fold changes were positively correlated with serum calcium changes, suggesting that c-miR-21-5p was a useful biomarker for bone resorption in an unloaded condition (67). It is difficult to speculate on the underlying mechanisms for these correlations since we did not measure bone formation markers or target genes in this study.

C-miRNAs have potential to serve as biomarkers for age-related diseases such as osteoporosis and sarcopenia (3, 11, 68). There are several advantages for using c-miRNAs as biomarkers compared to other bone markers; miRNAs are stable in the blood, able to withstand multiple freeze-thaw cycles, and measurement with qPCR is a reliable and well-established method for molecular diagnostics (3). Pre-analytical factors such as stress, drugs, sleep, alcohol, smoking, and diet can affect miRNA expression (69) but can be controlled by the research design. To be useful as biomarkers for osteoporosis, c-miRNAs need to correlate with, and accurately predict, bone status. In a previous study, we reported that resting expression of c-miR-21-5p and -23a-3p were negatively correlated with hip and spine aBMD in elderly postmenopausal women (70). In this study, baseline c-miR-21-5p and -148a-3p were the predominant miRNAs showing significant negative correlations with tibia BMC, bone area, and bone strength variables, suggesting these miRNAs have potential as biomarkers for tibia bone status in postmenopausal women. In contrast, c-miR-23a-3p was negatively correlated only with total hip aBMD and tibia cortical area, and c-miR-133a-3p was positively correlated with TRAP5b and spine aBMD. Circulating miRNAs, including miR-133a-3p, also have shown promise as biomarkers for the muscle-related disease, Duchenne Muscular Dystrophy (71), and may have further utility for detecting sarcopenia. We did not find any significant correlations between the selected c-miRNAs and muscle variables. In contrast, Halper et al. (72) found that miR-21-5p had a low positive correlation with handgrip strength in 90 elderly women 65-92 years. The study mainly differed from the current by measuring absolute instead of relative expression and having an older mean population.

There are limitations to this study. The participants were mostly very physically active according to their IPAQ scores and may not reflect responses for sedentary or moderately active elderly postmenopausal women. This cohort was also fairly homogeneous in terms of bone and muscle status, with most of the participants meeting the T-score criteria for osteopenia or osteoporosis (48), and only two participants being classified as sarcopenic according to EWGSOP guidelines (47). We did not include a control day in this study, which would be helpful for determining circadian rhythms in the c-miRNAs (73). However, we did control for time of day and diet as all exercise tests were conducted in the morning (8:00-10:00 am) in a fasted condition, and the 24- and 48-hour blood draws were obtained at the same time of the morning as the exercise days. Another limitation is the miRNA assessment used. Some studies choose to include a discovery step to determine which miRNAs are expressed in their study population. This step is then repeated in a separate cohort to valid the miRNAs expressed. Due to the high cost of this approach, we instead chose to only target specific miRNAs that have been consistently and more frequently expressed in the literature and are involved in the regulation of bone metabolism. Normalization of miRNAs was limited to a single spike-in RNA control; while this method is acceptable for MIQE guidelines (42), there are assumptions during RNA isolation and cDNA synthesis that may have been violated (74). Inclusion of a larger number of miRNAs or measurement of endogenous controls would have provided the ability to use global mean normalization or geometric mean (74) and may reduce variability in relative expression.

## Conclusions

Our findings suggest that whole-body vibration is a sufficient mechanical stimulus for altering c-miR-21-5p expression, whereas a high intensity resistance exercise protocol did not elicit significant c-miRNA responses in postmenopausal women. C-miRNAs may also serve as biomarkers to track the progression of adaptations from exercise stimuli, though more studies are needed to determine which miRNAs are sensitive enough to express changes and have targets affecting tissues of interest (28, 31). The few exercise studies with miRNA analysis in older populations have focused on men, therefore, more research is warranted focusing on the c-miRNA responses in postmenopausal women.

## Data availability statement

The data used to support the findings of this study are restricted by the University of Oklahoma IRB 709 in order to protect participant privacy. Data may be available in aggregate form from the corresponding author upon request.

## Ethics statement

The studies involving human participants were reviewed and approved by University of Oklahoma Health Sciences Center Institutional Review Board. The patients/participants provided their written informed consent to participate in this study.

## Author contributions

SB wrote the first draft of the manuscript, contributed to the conception and design of the study, conducted the data collection, performed the bone marker assays, and performed the data analyses for all dependent variables. RM revised the manuscript and assisted with data collection and data analyses. MN revised the manuscript and assisted with data collection and the bone marker assays. CB, JMK, and MB all revised the manuscript and contributed to the conception, design, and data analyses of the study. DB revised the manuscript, contributed to the conception and design of the study, supervised the data collection and the bone marker assays and contributed to the data analyses. All authors contributed to the article and approved the submitted version.

## Funding

This study was funded by research grants from the Department of Health and Exercise Science and the Graduate College at the University of Oklahoma, and from the Central States Chapter of the American College of Sports Medicine. Financial support was provided by the University of Oklahoma Libraries’ Open Access Fund. 

## Acknowledgments

The authors express sincere gratitude to the participants, who graciously donated their time for this study. We also wish to thank Cameron Combs and Alison Balderas for their assistance with the data collection.

## Conflict of interest

The authors declare that the research was conducted in the absence of any commercial or financial relationships that could be construed as a potential conflict of interest.

## Publisher’s note

All claims expressed in this article are solely those of the authors and do not necessarily represent those of their affiliated organizations, or those of the publisher, the editors and the reviewers. Any product that may be evaluated in this article, or claim that may be made by its manufacturer, is not guaranteed or endorsed by the publisher.

## Glossary

**Table:**

ASMM	appendicular skeletal muscle mass
aBMD	Areal bone mineral density
BMP	Bone Morphogenetic Protein
BMPR/BMPR2	BMP receptor/type II
BPAQ	Bone-specific Physical Activity Questionnaire
BSI	bone strength index
cmiRNA	circulating microRNA
CTX-I	C-telopeptide of Type I collagen cross-links
CXCL11 C-X-C	Motif Chemokine Ligand 11
DXA	dual energy x-ray absorptiometry
eIF4E3	Eukaryotic translation initiation factor 4E family member 3
FASL	Fas Ligand
FC	fold change
HCT	haematocrit
IPAQ	International Physical Activity Questionnaire
KDM6B	Lysine Demethylase 6B
MAPK	Mitogen-Activated Protein Kinase
miRNA	microRNA
c-miRNA	circulating miRNA
KDM6B	Lysine Demethylase 6B
MAFB	V-maf musculoaponeurotic fibrosarcoma oncogene homolog B
MAFbx	Muscle Atrophy F-box
MCSA	Muscle cross-sectional area
MMP13	Matrix Metalloproteinase 13
mRNA	messenger RNA
MuRF1	Muscle RING Finger 1
Nt	nucleotides
PDCD4	Programmed Cell Death Protein 4
PDCD10	Programmed Cell Death Protein 10
pQCT	peripheral quantitative computed tomography
pSSI	strength-strain index
% PVΔ	percent plasma volume change;
RE	resistance exercise
1 RM	One repetition maximum
RMS CV%	root mean square coefficient of variation percent
RNA	Ribonucleic Acid
ROCK1	rho associated coiled-coil containing protein kinase1
RUNX2	Runt-Related Transcription Factor 2
SMAD1	Small Mothers Against Decapentaplegic 1
SMAD3	Small Mothers Against Decapentaplegic 3
SMAD7	Small Mothers Against Decapentaplegic 7
SRF	serum response factor
TGF-b	Transforming Growth Factor-Beta
TRAP5b	Tartrate-resistant acid phosphatase 5b
vBMD	volumetric bone mineral density
WBV	whole-body vibration
Wnt	Wingless-Type MMTV Integration Site Family
YY1	Yin and Yang 1
