# Supplementary material for: Circulating microRNA responses to acute whole-body vibration and resistance exercise in postmenopausal women
Source: Front Endocrinol (Lausanne). 2022 Nov 11;13:1038371. doi: 10.3389/fendo.2022.1038371 (PMC9692005; doi:10.3389/fendo.2022.1038371)
Supplement: Supplementary file 1 [file DataSheet_1.docx]

Supplementary Table 1. Cellular Regulatory Effects of Candidate miRNAs

| MiRNA | Target Gene | Biological Effect |
| --- | --- | --- |
| miR-21-5p  Bone | SMAD7  PDCD4 | Promote OB differentiation  Promote OC differentiation |
| Muscle | PDCD10, YY1  EIF4E3 | Promote muscle atrophy |
| miR-23a-3p  Bone | RUNX2 | Suppress OB differentiation |
| Muscle | MAFbx, MuRF1 | Inhibit muscle atrophy |
| miR-133a-3p  Bone | RUNX2  CXCL11 | Suppress OB differentiation  Promote OC differentiation |
| Muscle | SRF | Promote myoblast cell proliferation |
| miR-148a-3p  Bone | KDM6B  MAFB | Suppress OB differentiation  Promote OC differentiation |
| Muscle | ROCK1 | Promote myogenic differentiation |

OB - osteoblast; OC - osteoclast; SMAD7 - Small Mothers Against Decapentaplegic 7; PDCD4 - Programmed Cell Death Protein 4; PDCD10 - Programmed Cell Death Protein 10; YY1 – Yin and Yang 1; EIF4E3 - Eukaryotic Translation Initiation Factor 4E Family Member 3; RUNX2- Runt-related Transcription Factor 2; MAFbx - Muscle Atrophy F-box; MuRF1 - Muscle RING Finger 1; CXCL11 - C-X-C Motif Chemokine Ligand 11; SRF - Serum Response Factor; KDM6B- Lysine Demethylase 6B; MAFB - V-maf Musculoaponeurotic Fibrosarcoma Oncogene Homolog B; ROCK1 - Rho associated Coiled-coil Containing Protein Kinase 1

Modified from [7, 40, 55]

Supplementary Fig. 1. Study visits


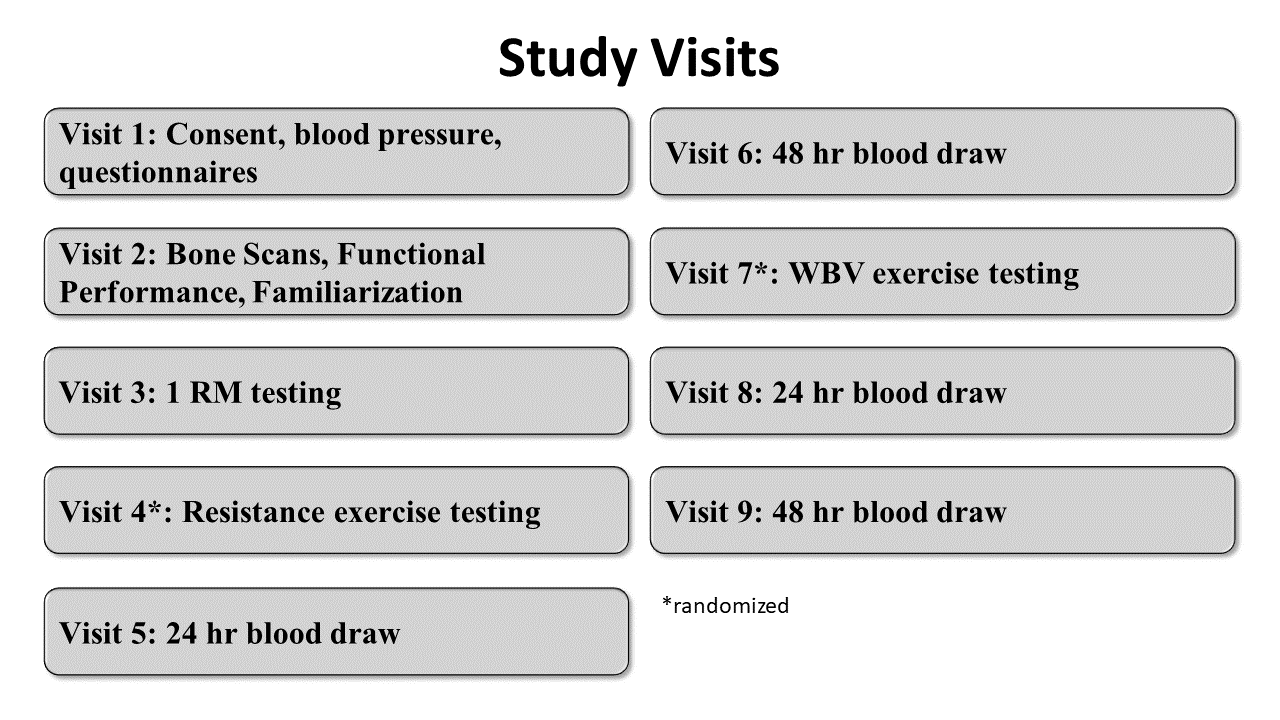


Supplementary Fig. 2. Blood sampling timeline for the resistance exercise (RE) (panel A) and whole-body vibration (WBV) (panel B) protocols
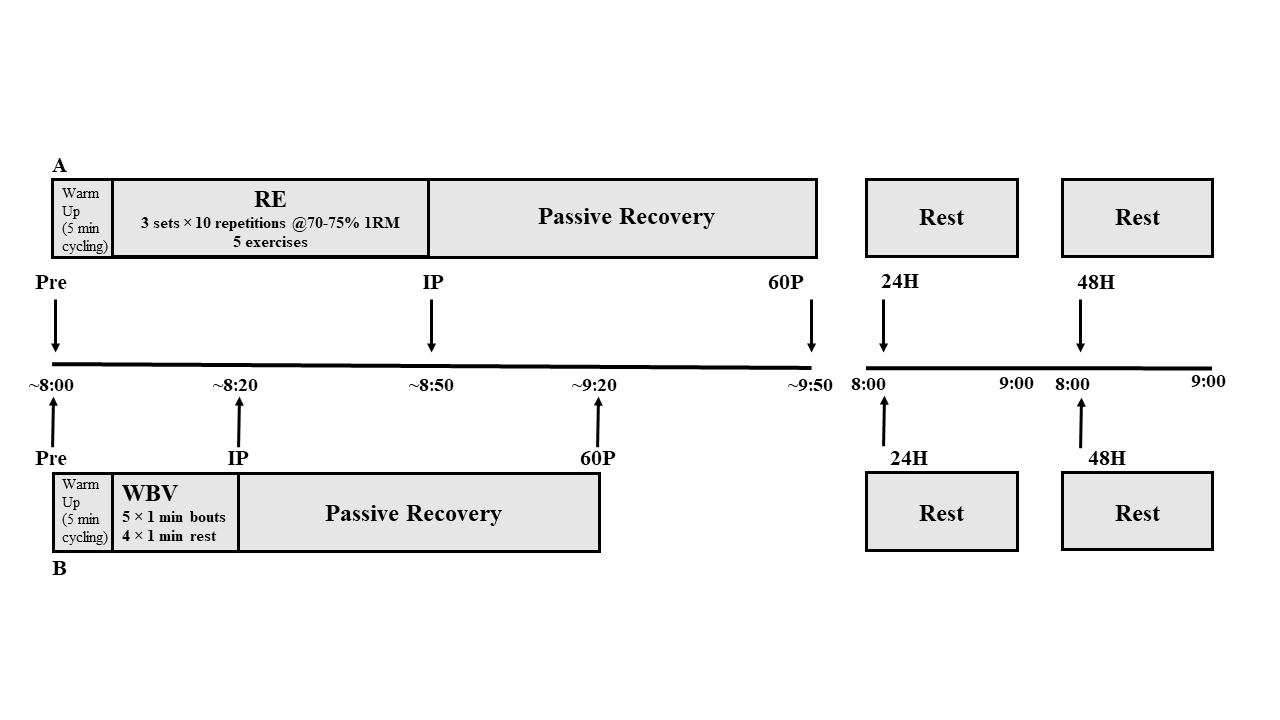


Supplementary Fig 3. Quality Control for miRNA spike-in


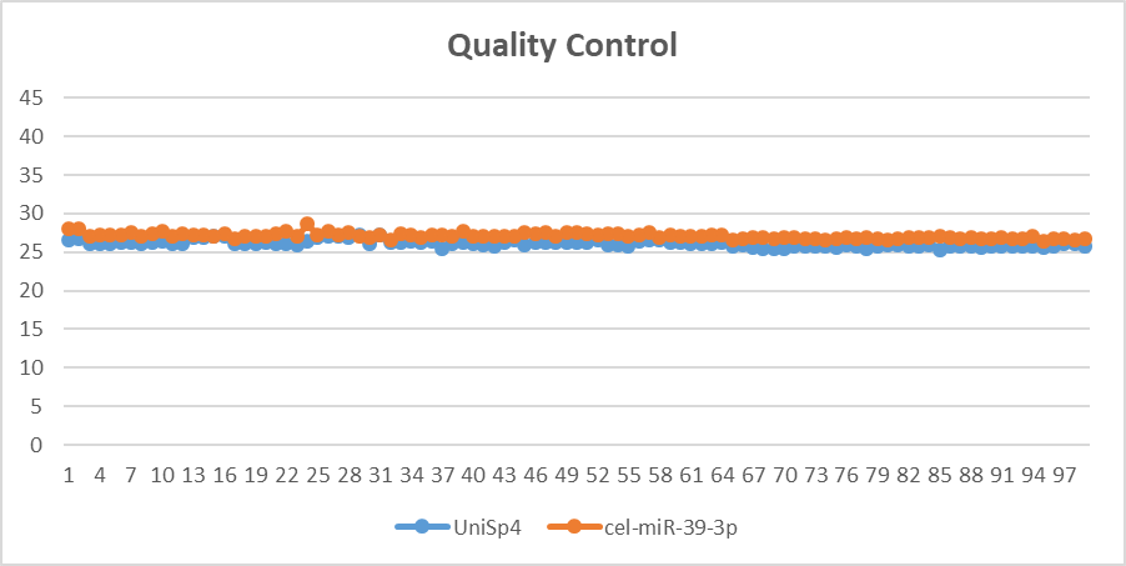


Supplementary Table 2. Muscular Strength Measures (n=10) (means ± SD)

| Leg Press (kg) | 81.6 | ± | 12.9 |
| --- | --- | --- | --- |
| Shoulder Press (kg) | 26.6 | ± | 5.1 |
| Lat Pulldown (kg) | 29.4 | ± | 6.3 |
| Leg Extension (kg) | 29.6 | ± | 7.8 |
| Right Hip Adduction (kg) | 33.1 | ± | 3.3 |
| Left Hip Adduction (kg) | 32.5 | ± | 3.7 |

Supplementary Table 3. Total Body, Lumbar Spine and Dual Hip aBMD (n=10) (means ± SD)

| Total Body aBMD (g/cm^2^) | 1.083 | ± | 0.073 |
| --- | --- | --- | --- |
| Total Body T-score | 0.02 | ± | 0.72 |
| L1-L4 aBMD (g/cm^2^) | 1.094 | ± | 0.117 |
| L1-L4 T-score | -0.71 | ± | 0.98 |
| Dominant Hip |  |  |  |
| Femoral Neck aBMD (g/cm^2^) | 0.826 | ± | 0.097 |
| Femoral Neck T-score | -1.52 | ± | 0.71 |
| Trochanter aBMD (g/cm^2^) | 0.707 | ± | 0.060 |
| Trochanter T-score | -1.25 | ± | 0.51 |
| Total Hip aBMD (g/cm^2^) | 0.867 | ± | 0.062 |
| Total Hip T-score | -1.1 | ± | 0.47 |
| Non-Dominant Hip |  |  |  |
| Femoral Neck aBMD (g/cm^2^) | 0.837 | ± | 0.096 |
| Femoral Neck T-score | -1.45 | ± | 0.67 |
| Trochanter aBMD (g/cm^2^) | 0.715 | ± | 0.080 |
| Trochanter T-score | -1.19 | ± | 0.70 |
| Total Hip aBMD (g/cm^2^) | 0.874 | ± | 0.059 |
| Total Hip T-score | -1.05 | ± | 0.45 |
| aBMD - Areal Bone Mineral Density; L1-L4 – lumbar spine vertebrae 1-4 | | | |

Supplementary Table 4. Tibia Bone Variables (means ± SD)

|  | (n=10) | | |
| --- | --- | --- | --- |
| 4% Tibia |  |  |  |
| Total |  |  |  |
| BMC (mg/mm) | 271.9 | ± | 39.9 |
| vBMD (g/cm³) | 270.6 | ± | 28.7 |
| Area (mm²) | 1006.11 | ± | 117.69 |
| BSI (mg²/mm⁴) | 112.3 | ± | 6.6 |
| Trabecular |  |  |  |
| BMC (mg/mm) | 197.3 | ± | 36.2 |
| vBMD (g/cm³) | 236.52 | ± | 30.98 |
| Area (mm²) | 833.82 | ± | 103.01 |
| BSI (mg²/mm⁴) | 74.2 | ± | 16.2 |
| Peri C (mm) | 47.4 | ± | 13.5 |
| 38% Tibia |  |  |  |
| Total |  |  |  |
| BMC (mg/mm) | 294.7 | ± | 26.8 |
| vBMD (g/mm³) | 869.83 | ± | 69.53 |
| Area (mm²) | 340.51 | ± | 39.87 |
| Cortical |  |  |  |
| BMC (mg/mm) | 278.92 | ± | 24.08 |
| vBMD (g/mm³) | 1160.19 | ± | 20.60 |
| Area (mm²) | 240.61 | ± | 22.78 |
| Thickness (mm) | 4.82 | ± | 0.52 |
| Peri C (mm) | 65.3 | ± | 3.8 |
| Endo C (mm) | 35.0 | ± | 5.5 |
| Ipolar (mm⁴) | 18510.2 | ± | 3486.7 |
| pSSI (mm³) | 1354.2 | ± | 204.6 |
| 66% Tibia |  |  |  |
| Total |  |  |  |
| BMC (mg/mm) | 321.3 | ± | 33.7 |
| vBMD (g/mm³) | 647.79 | ± | 65.41 |
| Area (mm²) | 500.70 | ± | 71.06 |
| Cortical |  |  |  |
| BMC (mg/mm) | 283.2 | ± | 30.7 |
| vBMD (g/mm³) | 1102.16 | ± | 26.23 |
| Area (mm²) | 256.85 | ± | 26.72 |
| Thickness (mm) | 3.85 | ± | 0.43 |
| Peri C (mm) | 79.14 | ± | 5.6 |
| Endo C (mm) | 54.9 | ± | 7.1 |
| Ipolar (mm⁴) | 33494.0 | ± | 6568.1 |
| pSSI (mm³) | 1974.6 | ± | 327.4 |
| IMAT (mm²) | 1554.1 | ± | 359.5 |
| Muscle CSA (mm²) | 6151.3 | ± | 1121.0 |
| Muscle Density (mg/cm^3^) | 75.8 | ± | 2.5 |

BMC - Bone Mineral Content; vBMD - volumetric Bone Mineral Density; BSI - Bone Strength Index; Peri C - Periosteal Circumference; Endo C - Endosteal Circumference; Ipolar - Polar Moment of Inertia; pSSI - Strength-Strain Index; IMAT - Intramuscular Adipose Tissue; CSA – Cross-sectional Area

Supplementary Table 5. Pearson Correlation Matrix Between Pre-Exercise^a^ Circulating miRNA Expression and Bone Characteristics (n=10)

| Bone Variable | miR-21-5p | miR-23a-3p | miR-133a-3p | miR-148a-3p |
| --- | --- | --- | --- | --- |
| TRAP5b Pre | 0.191 | 0.161 | 0.758* | 0.278 |
| L1-L4 aBMD | -0.093 | -0.104 | 0.666* | -0.043 |
| L Total Hip aBMD | -0.411 | -0.642* | 0.163 | -0.443 |
| 4% Tibia Trab BMC | -0.658* | -0.534 | 0.409 | -0.594 |
| 4% Tibia Trab Area | -0.717* | -0.61 | -0.012 | -0.716* |
| 4% Tibia Total BSI | -0.692* | -0.599 | 0.0133 | -0.702* |
| 38% Tibia Cort BMC | -0.809** | -0.624 | -0.133 | -0.811** |
| 38% Tibia Cort vBMD | 0.142 | 0.324 | 0.055 | 0.414 |
| 38% Tibia Cort Area | -0.770** | -0.639* | -0.126 | -0.821** |
| 38% Tibia Ipolar | -0.437 | -0.365 | -0.35 | -0.694* |
| 38% Tibia pSSI | -0.472 | -0.362 | -0.399 | -0.666* |
| 66% Tibia Cort BMC | -0.877** | -0.511 | 0.188 | -0.637* |
| 66% Tibia Cort Area | -0.862** | -0.522 | 0.173 | -0.635* |
| 66% Tibia Peri C | -0.443 | -0.481 | -0.516 | -0.696* |
| 66% Tibia Endo C | -0.156 | -0.33 | -0.626 | -0.524 |
| 66% Tibia Ipolar | -0.660* | -0.564 | -0.352 | -0.779** |
| 66% Tibia pSSI | -0.727* | -0.557 | -0.263 | -0.768** |
| *p<0.05 **p<0.01; ^a^ averaged over both exercise days; TRAP5b - Tartrate-resistant acid phosphatase 5b; aBMD - areal Bone Mineral Density; L1-L4 - Lumbar spine 1-4; L - Left; Trab- Trabecular; BMC - Bone Mineral Content; vBMD - volumetric Bone Mineral Density; BSI- Bone Strength Index; Cort - Cortical;; Ipolar - Polar Moment of Inertia; pSSI- Strength-Strain Index; Peri C- Periosteal Circumference; Endo C- Endosteal Circumference | | | | |

Supplementary Table 6. Circulating miRNA Relative Expression for Whole-body Vibration (WBV) Unadjusted and Adjusted for Plasma Volume Changes (means ± SE)

|  | Relative Expression (n=10) | | | | | | | | | | Fold Change (vs. Pre) | | | |
| --- | --- | --- | --- | --- | --- | --- | --- | --- | --- | --- | --- | --- | --- | --- |
| miRNA | Pre | | IP | | 60P | | 24H^a^ | | 48H | | IP | 60P | 24H^a^ | 48H |
| miR-21-5p | 1.22 | ± 0.22 | 1.22 | ± 0.26 | 1.61 | ± 0.31 | 0.80 | ± 0.16**^*^** | 0.94 | ± 0.19 | 0.94 | 1.31 | 0.75 | 0.82 |
| Adj21 |  |  | 1.04 | ± 0.25 | 1.43 | ± 0.29 |  |  |  |  | 0.89 | 1.16 |  |  |
| miR-23a-3p | -0.14 | ± 0.27 | -0.03 | ± 0.28 | 0.26 | ± 0.27 | -0.38 | ± 0.15 | -0.20 | ± 0.27 | 1.07 | 1.32 | 0.85 | 0.96 |
| Adj23 |  |  | -0.21 | ± 0.28 | 0.08 | ± 0.28 |  |  |  |  | 0.99 | 1.19 |  |  |
| miR-133a-3p | -8.03 | ± 0.43 | -7.31 | ± 0.36 | -7.35 | ± 0.33 | -7.61 | ± 0.40 | -7.70 | ± 0.42^b^ | 1.65 | 1.60 | 1.33 | 1.10^b^ |
| Adj133 |  |  | -7.48 | ± 0.37 | -7.53 | ± 0.98 |  |  |  |  | 1.46 | 1.41 |  |  |
| miR-148a-3p | -3.01 | ± 0.24 | -2.91 | ± 0.30 | -2.51 | ± 0.38 | -3.35 | ± 0.24 | -3.31 | ± 0.43 | 1.08 | 1.42 | 0.79 | 0.81 |
| Adj148 |  |  | -3.08 | ± 0.30 | -2.69 | ± 0.36 |  |  |  |  | 0.95 | 1.25 |  |  |
| ^a^ n=9 for this time point; ^b^ n=8 for this time point; ^*^ p<0.05 vs. 60P; Adj-adjusted for plasma volume shifts; Pre – pre-exercise; IP – immediately post-exercise; 60P – 60 minutes post-exercise; 24H – 24 hours post-exercise; 48H – 48 hours post-exercise | | | | | | | | | | | | | | |

Supplementary Table 7. Circulating miRNA Relative Expression for Resistance Exercise (RE) Unadjusted and Adjusted for Plasma Volume Changes (means ± SE)

|  | Relative Expression (n=10) | | | | | | | | | | Fold Change (vs. Pre) | | | |
| --- | --- | --- | --- | --- | --- | --- | --- | --- | --- | --- | --- | --- | --- | --- |
| miRNA | Pre | | IP | | 60P | | 24H^a^ | | 48H | | IP | 60P | 24H^a^ | 48H |
| miR-21-5p | 1.14 | ± 0.24 | 1.17 | ± 0.19 | 1.26 | ± 0.31 | 1.07 | ± 0.26 | 0.76 | ± 0.22 | 1.03 | 1.04 | 1.04 | 0.70 |
| Adj21 |  |  | 0.71 | ± 0.21 | 1.24 | ± 0.30 |  |  |  |  | 0.75 | 1.04 |  |  |
| miR-23a-3p | -0.29 | ± 0.23 | -0.13 | ± 0.18 | -0.08 | ± 0.20 | -0.27 | ± 0.23 | -0.32 | ± 0.18 | 1.12 | 1.12 | 1.04 | 0.90 |
| Adj23 |  |  | -0.59 | ± 0.20 | -0.09 | ± 0.21 |  |  |  |  | 0.82 | 1.13 |  |  |
| miR-133a-3p | -7.75 | ± 0.38 | -7.82 | ± 0.58 | -7.47 | ± 0.32 | -7.59 | ± 0.23 | -7.90 | ± 0.43^b^ | 0.95 | 1.54 | 1.33 | 0.79^b^ |
| Adj133 |  |  | -8.28 | ± 0.56 | -7.48 | ± 0.39 |  |  |  |  | 0.69 | 1.54 |  |  |
| miR-148a-3p | -2.98 | ± 0.27 | -3.05 | ± 0.17 | -2.98 | ± 0.33 | -3.31 | ± 0.31 | -3.18 | ± 0.26 | 0.95 | 1.00 | 0.93 | 0.87 |
| Adj148 |  |  | -3.50 | ± 0.18 | -2.99 | ± 0.34 |  |  |  |  | 0.69 | 0.98 |  |  |
| ^a^ n=9 for this time point; ^b^ n=8 for this time point; Adj-adjusted for plasma volume shifts; Pre – pre-exercise; IP – immediately post-exercise; 60P – 60 minutes post-exercise; 24H – 24 hours post-exercise; 48H – 48 hours post-exercise | | | | | | | | | | | | | | |
